# Ethyl 8-(4-nitro­phen­yl)imidazo[1,2-*a*]pyridine-7-carboxyl­ate

**DOI:** 10.1107/S1600536810047938

**Published:** 2010-11-24

**Authors:** Gui-Yun Duan, Yu-Juan Zhang, Ben-Qian Hao

**Affiliations:** a College of Pharmaceutical Sciences, Taishan Medical University, Tai an 271016, People’s Republic of China

## Abstract

In the title compound, C_16_H_13_N_3_O_4_, the imidazo[1,2-*a*]pyridine and benzene rings make a dihedral angle of 56.21 (2)°. The crystal packing is stabilized by weak π–π stacking inter­actions [centroid–centroid distances = 3.787 (2) Å] and C—H⋯O inter­molecular hydrogen-bonding inter­actions.

## Related literature

For applications of imidazo[1,2-*a*]pyridine-containing compounds, see: Jia *et al.* (2010 ▶).
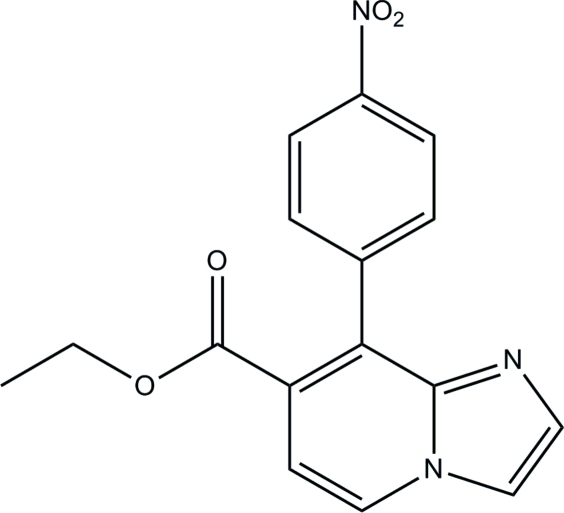


## Experimental

### 

#### Crystal data

C_16_H_13_N_3_O_4_
*M* *_r_* = 311.29
Monoclinic, 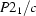

*a* = 8.189 (4) Å
*b* = 15.821 (8) Å
*c* = 11.884 (6) Å
β = 105.380 (8)°
*V* = 1484.7 (13) Å^3^
*Z* = 4
Mo *K*α radiation
μ = 0.10 mm^−1^
*T* = 273 K
0.26 × 0.19 × 0.13 mm

#### Data collection

Bruker SMART CCD area-detector diffractometer
Absorption correction: multi-scan (*SADABS*; Sheldrick, 1996 ▶) *T*_min_ = 0.974, *T*_max_ = 0.987
7569 measured reflections
2618 independent reflections
1965 reflections with *I* > 2σ(*I*)
*R*_int_ = 0.023

#### Refinement

*R*[*F* ^2^ > 2σ(*F* ^2^)] = 0.042
*wR*(*F* ^2^) = 0.122
*S* = 1.38
2618 reflections
209 parameters
H-atom parameters constrained
Δρ_max_ = 0.18 e Å^−3^
Δρ_min_ = −0.16 e Å^−3^

### 

Data collection: *SMART* (Bruker, 1998 ▶); cell refinement: *SAINT* (Bruker, 1999 ▶); data reduction: *SAINT*; program(s) used to solve structure: *SHELXS97* (Sheldrick, 2008 ▶); program(s) used to refine structure: *SHELXL97* (Sheldrick, 2008 ▶); molecular graphics: *SHELXTL* (Sheldrick, 2008 ▶); software used to prepare material for publication: *SHELXTL*.

## Supplementary Material

Crystal structure: contains datablocks global, I. DOI: 10.1107/S1600536810047938/hg2751sup1.cif

Structure factors: contains datablocks I. DOI: 10.1107/S1600536810047938/hg2751Isup2.hkl

Additional supplementary materials:  crystallographic information; 3D view; checkCIF report

## Figures and Tables

**Table 1:** Hydrogen-bond geometry (Å, °)

*D*—H⋯*A*	*D*—H	H⋯*A*	*D*⋯*A*	*D*—H⋯*A*
C9—H9*B*⋯O3^i^	0.97	2.59	3.295 (3)	130

Symmetry code: (i) 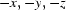.

**Fig. 1.**
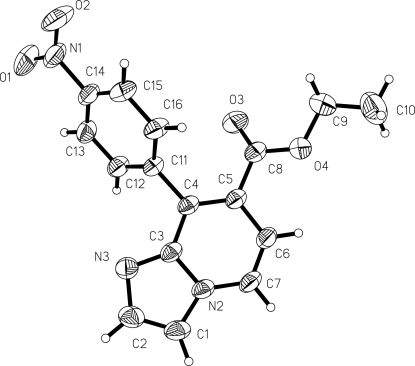
View of the title compound (I), with displacement ellipsoids drawn at the 40% probability level.

## supplementary crystallographic information

### Comment

The imidazo[1,2-*a*]pyridines (IP) have attracted considerable attention because of their wide range of pharmacological activities such as antiviral, antibacterial, antifungal, antiulcer, and anti-inflammatory behavior (Jia *et al.*, 2010). Drugs containing imidazo[1,2-*a*]pyridines such as Alpidem, Zolpidem, Necopidem, Olprinone, Divalpon and Zolimidine are currently available on the market. In continuation of our work in this direction, we report here the crystal structure of the title compound, (I).

The title compound, C_16_H_13_N_3_O_4_, the imidazo[1,2-*a*]pyridine ring (N2/N3/C1—C7) and benzene ring (C11—C16) make a dihedral angles of 56.21 (2) °. π—π interactions are indicated by the short distance (*Cg*1···*Cg*2 distance of 3.787 (2) Å, symmetry code: *x*,1/2 - *y*,-1/2 + *z*) between the centroids of the pyridine ring (N2/C3—C7) (*Cg*1) and benzene ring C11—C16 (*Cg*2) (Table 1). There are weaker C—H···O intermolecular interactions, which stabilize the structure (Table 1).

### Experimental

To a 50-ml round-bottomed flask were added ethyl 4-bromobut-2-enoate (1.20 mmol), (1*H*-imidazol-2-yl)(4-nitrophenyl)methanone (1.00 mmol), potassium carbonate (0.283 g, 2.05 mmol) and dry DMF (10 ml). The mixture was stirred at rt for 3 h and then filtered. The filtrate was poured into water (100 ml) and extracted with CH_2_Cl_2_ (three times per 30 ml). The combined extracts were washed with water, dried over anhydrous MgSO_4_ and filtered, and the solvent was removed by rotary evaporation. The crude product were purified by column chromatography. Crystals of (I) suitable for X-ray diffraction was obtained by slow evaporation of a solution of the product in ethyl acetate at room temperature for 2 d.

### Refinement

H atoms were positioned geometrically and refined using a riding model, with C—H = 0.93 or 0.97Å and with *U*_iso_(H) = 1.2*U*_eq_(C) and 1.5*U*_eq_(C) for methyl H atoms.

### Crystal data

**Table:**

C_16_H_13_N_3_O_4_	*F*(000) = 648
*M**_r_* = 311.29	*D*_x_ = 1.393 Mg m^−^^3^
Monoclinic, *P*2_1_/*c*	Mo *K*α radiation, λ = 0.71073 Å
Hall symbol: -P 2ybc	Cell parameters from 2224 reflections
*a* = 8.189 (4) Å	θ = 2.2–28.2°
*b* = 15.821 (8) Å	µ = 0.10 mm^−^^1^
*c* = 11.884 (6) Å	*T* = 273 K
β = 105.380 (8)°	Block, colorless
*V* = 1484.7 (13) Å^3^	0.26 × 0.19 × 0.13 mm
*Z* = 4	

### Data collection

**Table:**

Bruker SMART CCD area-detector diffractometer	2618 independent reflections
Radiation source: fine-focus sealed tube	1965 reflections with *I* > 2σ(*I*)
graphite	*R*_int_ = 0.023
phi and ω scans	θ_max_ = 25.0°, θ_min_ = 2.2°
Absorption correction: multi-scan (*SADABS*; Sheldrick, 1996)	*h* = −9→9
*T*_min_ = 0.974, *T*_max_ = 0.987	*k* = −18→14
7569 measured reflections	*l* = −13→14

### Refinement

**Table:**

Refinement on *F*^2^	Secondary atom site location: difference Fourier map
Least-squares matrix: full	Hydrogen site location: inferred from neighbouring sites
*R*[*F*^2^ > 2σ(*F*^2^)] = 0.042	H-atom parameters constrained
*wR*(*F*^2^) = 0.122	*w* = 1/[σ^2^(*F*_o_^2^) + (0.0514*P*)^2^] where *P* = (*F*_o_^2^ + 2*F*_c_^2^)/3
*S* = 1.38	(Δ/σ)_max_ = 0.014
2618 reflections	Δρ_max_ = 0.18 e Å^−^^3^
209 parameters	Δρ_min_ = −0.16 e Å^−^^3^
0 restraints	Extinction correction: *SHELXL97* (Sheldrick, 2008), Fc^*^=kFc[1+0.001xFc^2^λ^3^/sin(2θ)]^-1/4^
Primary atom site location: structure-invariant direct methods	Extinction coefficient: 0.018 (2)

### Special details

**Table:**

Geometry. All e.s.d.'s (except the e.s.d. in the dihedral angle between two l.s. planes) are estimated using the full covariance matrix. The cell e.s.d.'s are taken into account individually in the estimation of e.s.d.'s in distances, angles and torsion angles; correlations between e.s.d.'s in cell parameters are only used when they are defined by crystal symmetry. An approximate (isotropic) treatment of cell e.s.d.'s is used for estimating e.s.d.'s involving l.s. planes.
Refinement. Refinement of *F*^2^ against ALL reflections. The weighted *R*-factor *wR* and goodness of fit *S* are based on *F*^2^, conventional *R*-factors *R* are based on *F*, with *F* set to zero for negative *F*^2^. The threshold expression of *F*^2^ > σ(*F*^2^) is used only for calculating *R*-factors(gt) *etc*. and is not relevant to the choice of reflections for refinement. *R*-factors based on *F*^2^ are statistically about twice as large as those based on *F*, and *R*- factors based on ALL data will be even larger.

### Fractional atomic coordinates and isotropic or equivalent isotropic displacement parameters (Å^2^)

**Table:**

	*x*	*y*	*z*	*U*_iso_*/*U*_eq_	
O1	0.4896 (3)	0.09876 (13)	0.56705 (14)	0.1106 (7)	
O2	0.2458 (2)	0.15378 (13)	0.49165 (14)	0.1073 (7)	
O3	0.26198 (16)	0.02576 (9)	0.02277 (12)	0.0718 (4)	
O4	0.16766 (15)	0.07201 (9)	−0.15928 (11)	0.0664 (4)	
N1	0.3843 (3)	0.12901 (12)	0.48549 (16)	0.0745 (5)	
N2	0.72970 (16)	0.19702 (9)	−0.06373 (12)	0.0490 (4)	
N3	0.80707 (17)	0.22853 (10)	0.12673 (13)	0.0561 (4)	
C1	0.8789 (2)	0.24074 (12)	−0.04309 (17)	0.0604 (5)	
H1	0.9381	0.2550	−0.0972	0.073*	
C2	0.9223 (2)	0.25889 (13)	0.07191 (18)	0.0617 (5)	
H2	1.0193	0.2886	0.1096	0.074*	
C3	0.6901 (2)	0.19066 (11)	0.04239 (14)	0.0460 (4)	
C4	0.53881 (19)	0.14865 (10)	0.04674 (14)	0.0432 (4)	
C5	0.4368 (2)	0.11716 (11)	−0.05585 (14)	0.0456 (4)	
C6	0.4823 (2)	0.12748 (12)	−0.16214 (14)	0.0521 (5)	
H6	0.4108	0.1070	−0.2311	0.063*	
C7	0.6262 (2)	0.16622 (12)	−0.16469 (15)	0.0550 (5)	
H7	0.6555	0.1721	−0.2347	0.066*	
C8	0.2819 (2)	0.06715 (11)	−0.05705 (15)	0.0500 (4)	
C9	0.0130 (2)	0.02350 (15)	−0.17049 (19)	0.0762 (6)	
H9A	0.0405	−0.0349	−0.1484	0.091*	
H9B	−0.0509	0.0467	−0.1197	0.091*	
C10	−0.0864 (3)	0.02810 (19)	−0.2914 (2)	0.1084 (9)	
H10A	−0.1100	0.0862	−0.3130	0.163*	
H10B	−0.1909	−0.0019	−0.3003	0.163*	
H10C	−0.0238	0.0031	−0.3407	0.163*	
C11	0.4992 (2)	0.14378 (10)	0.16178 (13)	0.0444 (4)	
C12	0.6133 (2)	0.10865 (12)	0.25694 (14)	0.0534 (5)	
H12	0.7159	0.0879	0.2490	0.064*	
C13	0.5773 (2)	0.10392 (12)	0.36384 (15)	0.0576 (5)	
H13	0.6541	0.0801	0.4280	0.069*	
C14	0.4254 (3)	0.13518 (12)	0.37291 (15)	0.0551 (5)	
C15	0.3104 (2)	0.17153 (12)	0.28087 (17)	0.0606 (5)	
H15	0.2089	0.1931	0.2897	0.073*	
C16	0.3482 (2)	0.17554 (12)	0.17493 (15)	0.0551 (5)	
H16	0.2711	0.1999	0.1114	0.066*	

### Atomic displacement parameters (Å^2^)

**Table:**

	*U*^11^	*U*^22^	*U*^33^	*U*^12^	*U*^13^	*U*^23^
O1	0.1407 (15)	0.1542 (18)	0.0415 (9)	−0.0163 (13)	0.0320 (10)	0.0017 (10)
O2	0.1239 (15)	0.1407 (17)	0.0844 (12)	−0.0061 (12)	0.0754 (11)	−0.0159 (10)
O3	0.0773 (9)	0.0782 (10)	0.0643 (9)	−0.0097 (7)	0.0263 (7)	0.0181 (7)
O4	0.0542 (8)	0.0928 (11)	0.0517 (8)	−0.0126 (7)	0.0132 (6)	0.0050 (7)
N1	0.1072 (15)	0.0807 (13)	0.0470 (11)	−0.0257 (11)	0.0407 (11)	−0.0149 (9)
N2	0.0456 (8)	0.0610 (9)	0.0463 (9)	0.0069 (7)	0.0227 (7)	0.0053 (7)
N3	0.0464 (9)	0.0705 (11)	0.0539 (9)	0.0020 (7)	0.0180 (8)	−0.0055 (8)
C1	0.0479 (11)	0.0750 (13)	0.0668 (13)	0.0025 (9)	0.0298 (10)	0.0064 (10)
C2	0.0440 (10)	0.0723 (13)	0.0731 (14)	0.0006 (9)	0.0230 (10)	−0.0030 (10)
C3	0.0459 (10)	0.0546 (10)	0.0422 (10)	0.0116 (8)	0.0199 (8)	0.0030 (8)
C4	0.0446 (9)	0.0490 (10)	0.0405 (9)	0.0096 (7)	0.0193 (8)	0.0046 (7)
C5	0.0484 (9)	0.0523 (10)	0.0397 (10)	0.0070 (8)	0.0179 (8)	0.0038 (8)
C6	0.0534 (10)	0.0671 (12)	0.0383 (10)	0.0034 (9)	0.0166 (8)	0.0020 (8)
C7	0.0609 (11)	0.0726 (13)	0.0382 (10)	0.0078 (9)	0.0247 (9)	0.0057 (9)
C8	0.0550 (11)	0.0536 (11)	0.0463 (11)	0.0053 (8)	0.0221 (9)	−0.0001 (8)
C9	0.0588 (12)	0.0869 (16)	0.0848 (17)	−0.0171 (11)	0.0225 (11)	0.0004 (12)
C10	0.0882 (18)	0.117 (2)	0.102 (2)	−0.0324 (16)	−0.0065 (15)	0.0071 (16)
C11	0.0499 (10)	0.0496 (10)	0.0381 (9)	0.0030 (8)	0.0192 (8)	0.0013 (7)
C12	0.0544 (10)	0.0637 (12)	0.0454 (10)	0.0073 (9)	0.0186 (9)	0.0024 (8)
C13	0.0681 (12)	0.0668 (12)	0.0369 (10)	−0.0044 (10)	0.0120 (9)	0.0033 (8)
C14	0.0756 (13)	0.0571 (11)	0.0410 (10)	−0.0129 (10)	0.0302 (9)	−0.0087 (8)
C15	0.0675 (12)	0.0658 (12)	0.0605 (12)	0.0065 (10)	0.0380 (10)	−0.0009 (10)
C16	0.0573 (11)	0.0651 (12)	0.0500 (11)	0.0121 (9)	0.0269 (9)	0.0087 (9)

### Geometric parameters (Å, °)

**Table:**

O1—N1	1.212 (2)	C6—C7	1.336 (2)
O2—N1	1.221 (2)	C6—H6	0.9300
O3—C8	1.199 (2)	C7—H7	0.9300
O4—C8	1.324 (2)	C9—C10	1.453 (3)
O4—C9	1.456 (2)	C9—H9A	0.9700
N1—C14	1.467 (2)	C9—H9B	0.9700
N2—C7	1.362 (2)	C10—H10A	0.9600
N2—C1	1.368 (2)	C10—H10B	0.9600
N2—C3	1.387 (2)	C10—H10C	0.9600
N3—C3	1.331 (2)	C11—C12	1.379 (2)
N3—C2	1.368 (2)	C11—C16	1.382 (2)
C1—C2	1.349 (3)	C12—C13	1.380 (2)
C1—H1	0.9300	C12—H12	0.9300
C2—H2	0.9300	C13—C14	1.369 (3)
C3—C4	1.419 (2)	C13—H13	0.9300
C4—C5	1.376 (2)	C14—C15	1.367 (3)
C4—C11	1.488 (2)	C15—C16	1.375 (2)
C5—C6	1.418 (2)	C15—H15	0.9300
C5—C8	1.492 (2)	C16—H16	0.9300
			
C8—O4—C9	116.01 (15)	O4—C8—C5	111.70 (14)
O1—N1—O2	123.67 (18)	C10—C9—O4	108.03 (18)
O1—N1—C14	118.0 (2)	C10—C9—H9A	110.1
O2—N1—C14	118.4 (2)	O4—C9—H9A	110.1
C7—N2—C1	131.00 (15)	C10—C9—H9B	110.1
C7—N2—C3	122.27 (14)	O4—C9—H9B	110.1
C1—N2—C3	106.67 (15)	H9A—C9—H9B	108.4
C3—N3—C2	104.42 (15)	C9—C10—H10A	109.5
C2—C1—N2	105.78 (16)	C9—C10—H10B	109.5
C2—C1—H1	127.1	H10A—C10—H10B	109.5
N2—C1—H1	127.1	C9—C10—H10C	109.5
C1—C2—N3	112.23 (17)	H10A—C10—H10C	109.5
C1—C2—H2	123.9	H10B—C10—H10C	109.5
N3—C2—H2	123.9	C12—C11—C16	119.08 (15)
N3—C3—N2	110.90 (14)	C12—C11—C4	120.61 (15)
N3—C3—C4	130.12 (15)	C16—C11—C4	120.30 (14)
N2—C3—C4	118.97 (15)	C11—C12—C13	120.87 (16)
C5—C4—C3	117.92 (14)	C11—C12—H12	119.6
C5—C4—C11	124.50 (15)	C13—C12—H12	119.6
C3—C4—C11	117.56 (15)	C14—C13—C12	118.25 (17)
C4—C5—C6	120.44 (16)	C14—C13—H13	120.9
C4—C5—C8	121.03 (14)	C12—C13—H13	120.9
C6—C5—C8	118.43 (15)	C15—C14—C13	122.45 (16)
C7—C6—C5	120.98 (17)	C15—C14—N1	118.81 (18)
C7—C6—H6	119.5	C13—C14—N1	118.74 (19)
C5—C6—H6	119.5	C14—C15—C16	118.54 (17)
C6—C7—N2	119.39 (15)	C14—C15—H15	120.7
C6—C7—H7	120.3	C16—C15—H15	120.7
N2—C7—H7	120.3	C15—C16—C11	120.80 (17)
O3—C8—O4	123.14 (17)	C15—C16—H16	119.6
O3—C8—C5	125.15 (17)	C11—C16—H16	119.6
			
C7—N2—C1—C2	177.07 (17)	C9—O4—C8—C5	178.48 (15)
C3—N2—C1—C2	−0.19 (19)	C4—C5—C8—O3	−27.4 (3)
N2—C1—C2—N3	0.1 (2)	C6—C5—C8—O3	149.05 (18)
C3—N3—C2—C1	0.1 (2)	C4—C5—C8—O4	154.15 (15)
C2—N3—C3—N2	−0.17 (18)	C6—C5—C8—O4	−29.4 (2)
C2—N3—C3—C4	−179.10 (17)	C8—O4—C9—C10	−173.05 (19)
C7—N2—C3—N3	−177.32 (15)	C5—C4—C11—C12	125.59 (19)
C1—N2—C3—N3	0.23 (18)	C3—C4—C11—C12	−56.1 (2)
C7—N2—C3—C4	1.7 (2)	C5—C4—C11—C16	−55.3 (2)
C1—N2—C3—C4	179.30 (14)	C3—C4—C11—C16	122.98 (19)
N3—C3—C4—C5	177.88 (16)	C16—C11—C12—C13	0.9 (3)
N2—C3—C4—C5	−1.0 (2)	C4—C11—C12—C13	−179.97 (16)
N3—C3—C4—C11	−0.5 (3)	C11—C12—C13—C14	−0.1 (3)
N2—C3—C4—C11	−179.36 (13)	C12—C13—C14—C15	−0.9 (3)
C3—C4—C5—C6	−0.5 (2)	C12—C13—C14—N1	178.82 (16)
C11—C4—C5—C6	177.72 (15)	O1—N1—C14—C15	−177.96 (19)
C3—C4—C5—C8	175.81 (14)	O2—N1—C14—C15	2.7 (3)
C11—C4—C5—C8	−5.9 (2)	O1—N1—C14—C13	2.4 (3)
C4—C5—C6—C7	1.4 (3)	O2—N1—C14—C13	−176.97 (18)
C8—C5—C6—C7	−175.01 (16)	C13—C14—C15—C16	1.0 (3)
C5—C6—C7—N2	−0.7 (3)	N1—C14—C15—C16	−178.65 (17)
C1—N2—C7—C6	−177.78 (17)	C14—C15—C16—C11	−0.2 (3)
C3—N2—C7—C6	−0.9 (2)	C12—C11—C16—C15	−0.7 (3)
C9—O4—C8—O3	0.0 (3)	C4—C11—C16—C15	−179.87 (16)

### Hydrogen-bond geometry (Å, °)

**Table:**

*D*—H···*A*	*D*—H	H···*A*	*D*···*A*	*D*—H···*A*
C9—H9B···O3^i^	0.97	2.59	3.295 (3)	130

Symmetry codes: (i) −*x*, −*y*, −*z*.
